# Bacteria flying under the radar: linking a bacterial infection to colon carcinogenesis

**DOI:** 10.1186/1750-9378-9-31

**Published:** 2014-09-11

**Authors:** Jacqueline I Keenan, Frank A Frizelle

**Affiliations:** 1 Department of Surgery, University of Otago Christchurch, PO Box 4345, Christchurch 8140, New Zealand

**Keywords:** *Bacteroides fragilis*, Colorectal cancer, Host response, Bacterial persistence

## Abstract

The emergence of a link between *Helicobacter pylori* infection and an increased risk of gastric cancer has raised an awareness of a possible link between colonic microbiota and colorectal cancer. Pertubation of the colonic epithelium by toxin-producing strains of *Bacteroides fragilis* may increase the risk of premalignant transdifferentiation. However, like *H. pylori*, *B. fragilis* exhibit an ability to modulate the normal host response to infection. We speculate this may be an underappreciated risk factor in the genesis of colon carcinogenesis in individuals colonised with toxin-producing strains of *B. fragilis*.

## Letters to the Editor

### Gut bacteria and carcinogenesis

More than 90% of colorectal cancers are considered sporadic and being able to identify those who are at risk of developing this disease could reduce the number of colorectal cancer (CRC)-related deaths. Carcinogenesis is initiated when somatic changes induce irreversible DNA alterations that can persist indefinitely in cells and evidence is increasing of a role for bacterial species in this process, exemplified by chronic *Helicobacter pylori* infection and gastric cancer [1]. The mechanism(s) of carcinogenesis associated with *H. pylori* colonization remains an area of debate but childhood acquisition of infection is reportedly associated with increased risk [2] that is strengthened by the carriage of strains carrying genes that code for specific virulence factors. The *ca*g-pathogenicity island is associated with the severity of precancerous lesions [3] via increased secretion of the pro-inflammatory cytokine interleukin (IL)-8 and the production of reactive oxygen species (ROS) [4]. Strains that express the s1m1 variant of the VacA cytotoxin may increase risk via toxin-associated DNA damage [5]. The expression of cytokines and chemokines (such as IL-8) create an immune microenvironment with the potential to exacerbate toxin- and/or ROS-mediated DNA damage [6]. Thus, chronic inflammation may contribute to the hypermethylation of DNA that, in part, drives cells to become malignant.

### Enterotoxigenic *Bacteroides fragilis* and CRC

*Bacteroides fragilis* is a ubiquitous anaerobic Gram-negative bacterium that colonises the human colon. Transmission is likely intra-familial and once acquired infection is persistent, much like *H. pylori*. Some strains produce a heat-labile toxin and infection with enterotoxigenic *B. fragilis* (ETBF) is associated with diarrhoea in children, adults and livestock. *B. fragilis* toxin (BFT) reportedly stimulates secretion of pro-inflammatory cytokine IL-8 from cultured intestinal epithelial cells [7] and therefore has the potential to provoke persistent, occult inflammation *in vivo* that, in turn, likely exacerbates the induction of colitis and tumour formation in ETBF-colonised Min mice [8]. BFT also has the potential to perturb epithelial homeostasis through E-cadherin cleavage [9,10] that results in β-catenin nuclear signalling and colonic epithelial proliferation [11]. In mice, BFT expression is essential to disease pathogenesis [12]. Intriguingly, *H. pylori* CagA protein reportedly has a similar effect on gastric epithelial cell homeostasis [13]. Thus, like chronic *H. pylori* infection of the stomach, persistent ETBF infection may increase the risk of colon carcinogenesis via perturbation of apical-junctional complexes that increases the risk of premalignant transdifferentiation. This observation is supported by a recent Turkish study that found carriage of toxigenic, as opposed to non-toxigenic, *B. fragilis* increased in people with CRC [14].

### Bacterial persistence as a risk factor for carcinogenesis?

*H. pylori* and *B. fragilis* share a common physiology that may contribute to their ability to provoke sustained, low-grade inflammation. Gram-negative bacteria are defined by their outer membrane that is implicated in the host response to infection. Generally, it is the lipid A moiety (or endotoxin) of outer membrane-associated lipopolyosaccharide that signals the presence of these bacteria in host tissues [15] and genes that encode the host sensory mechanism that recognise lipid A have been found in almost all studied vertebrates [16]. However, the atypical structure of *H. pylori* lipid A [17] fails to activate the TLR4 signalling pathway and instead signals through TLR2 [18], making these bacteria less stimulatory to cells. *B. fragilis* lipid A shares the same structural differences as *H. pylori* lipid A [19]. In addition, some *B. fragilis* strains express a polysaccharide antigen A (PSA) that also signals through TLR2 [20]. These changes modulate the host response to infection, which fuels speculation that attenuated biological activity has the potential to contribute to their on-going infection of the human stomach and colon, respectively [21]. Accordingly, we speculate that this inherent ability to “fly under the radar” of the normal host response to infection may be an underappreciated factor that contributes to the persistent colonisation considered as key to the increased risk of colon carcinogenesis in individuals infected with toxin-producing strains of *B. fragilis*.

## Competing interests

We declare that we have no competing interests.

## Authors’ contributions

JK and FF were both involved in drafting and revising the manuscript. Both have read and approved the final manuscript.
